# Adverse Renal, Endocrine, Hepatic, and Metabolic Events during Maintenance Mood Stabilizer Treatment for Bipolar Disorder: A Population-Based Cohort Study

**DOI:** 10.1371/journal.pmed.1002058

**Published:** 2016-08-02

**Authors:** Joseph F. Hayes, Louise Marston, Kate Walters, John R. Geddes, Michael King, David P. J. Osborn

**Affiliations:** 1 Division of Psychiatry University College London, London, United Kingdom; 2 Primary Care and Population Health, University College London, London, United Kingdom; 3 Department of Psychiatry, University of Oxford, Oxford, United Kingdom; Massachusetts General Hospital, UNITED STATES

## Abstract

**Background:**

There is limited, poorly characterized information about adverse events occurring during maintenance treatment of bipolar disorder. We aimed to determine adverse event rates during treatment with lithium, valproate, olanzapine, and quetiapine.

**Methods and Findings:**

We conducted a propensity score adjusted cohort study using nationally representative United Kingdom electronic health records from January 1, 1995, until December 31, 2013. We included patients who had a diagnosis of bipolar disorder and were prescribed lithium (*n* = 2148), valproate (*n* = 1670), olanzapine (*n* = 1477), or quetiapine (*n* = 1376) as maintenance mood stabilizer treatment. Adverse outcomes were chronic kidney disease, thyroid disease, hypercalcemia, weight gain, hypertension, type 2 diabetes mellitus, cardiovascular disease, and hepatotoxicity. The propensity score included important demographic, physical health, and mental health predictors of drug treatment allocation. The median duration of drug treatment was 1.48 y (interquartile range 0.64–3.43). Compared to patients prescribed lithium, those taking valproate, olanzapine, and quetiapine had reduced rates of chronic kidney disease stage 3 or more severe, following adjustment for propensity score, age, and calendar year, and accounting for clustering by primary care practice (valproate hazard ratio [HR] 0.56; 95% confidence interval [CI] 0.45–0.69; *p* < 0.001, olanzapine HR 0.57; 95% CI 0.45–0.71; *p* < 0.001, quetiapine HR 0.62; 95% CI 0.47–0.80; *p* < 0.001). Hypothyroidism was reduced in those taking valproate (HR 0.60; 95% CI 0.40–0.89; *p* = 0.012) and olanzapine (HR 0.48; 95% CI 0.29–0.77; *p* = 0.003), compared to those taking lithium. Rates of new onset hyperthyroidism (valproate HR 0.24; 95% CI 0.09–0.61; *p* = 0.003, olanzapine HR 0.31; 95% CI 0.13–0.73; *p* = 0.007) and hypercalcemia (valproate HR 0.25; 95% CI 0.10–0.60; *p* = 0.002, olanzapine HR 0.32; 95% CI 0.14–0.76; *p* = 0.008, quetiapine HR 0.23; 95% CI 0.07–0.73; *p* = 0.013) were also reduced relative to lithium. However, rates of greater than 15% weight gain on valproate, olanzapine, and quetiapine were higher (valproate HR 1.62; 95% CI 1.31–2.01; *p* < 0.001, olanzapine HR 1.84; 95% CI 1.47–2.30; *p* < 0.001, quetiapine HR 1.67; 95% CI 1.24–2.20; *p* < 0.001) than in individuals prescribed lithium, as were rates of hypertension in the olanzapine treated group (HR 1.41, 95% CI 1.06–1.87; *p* = 0.017). We found no significant difference in rates of chronic kidney disease stage 4 or more severe, type 2 diabetes mellitus, cardiovascular disease, or hepatotoxicity. Despite estimates being robust following sensitivity analyses, limitations include the potential for residual confounding and ascertainment bias and an inability to examine dosage effects.

**Conclusions:**

Lithium use is associated with more renal and endocrine adverse events but less weight gain than commonly used alternative mood stabilizers. Risks need to be offset with the effectiveness and anti-suicidal benefits of lithium and the potential metabolic side effects of alternative treatment options.

## Abstract 2

In a study of data from the United Kingdom's primary care database, THIN, Joseph Hayes and colleagues report on adverse events associated with use of lithium and other maintenance treatments for bipolar disorder.

## Author Summary

**Why Was This Study Done?:**

- Although side effects of medications used for maintenance mood stabilizer treatment in bipolar disorder have been described, the relative risks of each drug are poorly understood.
- Randomised, controlled trials are unlikely to be able to give us this information because they include too few patients, with too short follow-up periods.
- We aimed to examine the rates of a number of adverse effects: renal failure, thyroid disease, hypercalcemia, hepatotoxicity, weight gain, type 2 diabetes mellitus, hypertension, and cardiovascular disease in patients taking the most commonly prescribed mood stabilizers in the United Kingdom: lithium, valproate, olanzapine, and quetiapine.

**What Did the Researchers Do and Find?:**

- We conducted a cohort study of individuals with bipolar disorder prescribed lithium (*n* = 2148), valproate (*n* = 1670), olanzapine (*n* = 1477), or quetiapine (*n* = 1376), accounting for key predictors of treatment assignment, such as mental and physical health history.
- We found that renal failure was more common in patients taking lithium (approximately 9 in 100 person years at risk) than in those taking the other drugs, but that rates of severe renal failure were similar in all groups (approximately 1 in 100 person years at risk).
- The rate of thyroid disease was elevated in people taking lithium, compared to valproate and olanzapine, but not quetiapine.
- The rate of hypercalcemia was increased with lithium compared to all other drugs.
- The rates of greater than 7% and greater than 15% weight gain were significantly higher in individuals taking valproate, olanzapine, or quetiapine compared to lithium (for example, the rate of greater than 15% weight gain in those taking olanzapine was 6 in 100 person years at risk).
- The rate of new onset hypertension was higher in those treated with olanzapine, compared to patients treated with lithium.
- We found no statistically significant differences in rates of type 2 diabetes mellitus, cardiovascular disease, or hepatotoxicity.

**What Do These Findings Mean?:**

- These findings highlight and quantify the relative risks of commonly used maintenance mood stabilizer treatments.
- The results for renal failure suggest that despite increasing rates of reduced renal function in those taking lithium compared to other drugs, severe renal failure is rare. We did not find differences in severe renal failure rates, which is in keeping with previous research. However, it is possible that this is due to the small number of people developing severe renal failure in each group.
- Although we did not find differences in rates of diabetes or cardiovascular disease, this may be due to relatively short follow-up times and the rarity of these adverse events. Weight gain is a significant risk factor for these health problems and was dramatically elevated in alternatives to lithium.
- Assiduous monitoring of patients prescribed lithium should ameliorate some risk associated with effects on renal physiology and endocrine systems. Calcium monitoring was rare in this representative cohort of patients with bipolar disorder in UK primary care, and this needs to be improved in clinical practice.
- The potential adverse effects of each of these drugs need to be balanced with their individual therapeutic benefits, and we hope that our findings will permit informed, collaborative discussions with patients.

## Introduction

Bipolar disorder (BPD) is a complex, recurrent, severe mental illness that affects over 350 million people worldwide [1]. Individuals with BPD will often require long-term drug treatment with the aim of preventing relapse or reccurrence [2]. Much of the evidence for maintenance medication comes from relatively short-term randomised controlled trials, which are then extrapolated to longer-term use [3]. However, this fails to take into account the potential longer-term adverse effects of the recommended medications. In 2014, the update of the United Kingdom National Institute for Health and Care Excellence (NICE) guidelines [3], a meta-analysis [4], and a network meta-analysis [5] all suggested that lithium should be seen as first-line monotherapy, whereas previous guidelines from around the world also recommended valproate, lamotrigine, carbamazepine, olanzapine, quetiapine, aripiprazole, oxcarbazepine, and risperidone injection [6,7]. Prescribing in the UK has reflected the previous NICE guidance for first-line treatment [8], with lithium, valproate, olanzapine, and quetiapine being the most frequently prescribed maintenance treatments [9].

A number of adverse effects of lithium have been identified since its use as a mood stabilizer became established in the 1970s [10], but it is only recently that they have begun to be characterised and quantified [11–15]. Lithium’s adverse effects include renal, thyroid, and parathyroid dysfunction. Lithium is also recognised to cause weight gain, but the risk of weight gain relative to other potential maintenance therapies has not been widely investigated [11]. Alternatives, such as second-generation antipsychotics and valproate, have been found to be obesogenic [16], especially olanzapine, which is the most commonly prescribed antipsychotic in BPD [9]. Weight gain is associated with a number of adverse events, such as hypertension, type 2 diabetes mellitus (T2DM), and cardiovascular disease (CVD) [17]. Valproate, olanzapine, and quetiapine are metabolized by the liver. Valproate has been found to be associated with a high risk of asymptomatic elevated transaminases and can cause idiosyncratic hepatic failure [15,18]. Olanzapine and quetiapine have also been associated with rare cases of hepatotoxicity [19–21]. Therefore, the balance of risks associated with maintenance mood stabilizer selection is not straightforward, and we are aware of no studies that make these comparisons across treatment options.

This study used a large electronic patient record database to compare rates of major recognised adverse outcomes amongst individuals prescribed lithium, valproate, olanzapine, or quetiapine for mood stabilization in BPD. The adverse events examined were chronic kidney disease (CKD), hypothyroidism, hyperthyroidism, hypercalcemia, weight gain, hypertension, T2DM, CVD, and hepatotoxicity [15,18].

## Methods

### Study Design

A population-based longitudinal cohort from January 1, 1995, to December 31, 2013.

### Setting

The Health Improvement Network (THIN) is a UK primary care database that contains anonymised patient information from routine clinical consultations [16]. The National Health Service (NHS) South-East Multicentre Research Ethics Committee approved THIN’s provision of anonymous patient data to researchers in 2003. Scientific approval for this study was obtained from the data provider’s Scientific Review Committee in March 2015.

THIN contained records of over 11 million people at the time of cohort extraction [22]. Included patients are broadly representative of the UK population, and physicians contributing data are representative in terms of consultation and prescribing statistics [23,24]. Approximately 98% of the UK population is registered with a primary care physician [25]. The incidence rate of BPD in THIN has been shown to be similar to European cohorts [26], and the validity of severe mental illness diagnoses held in primary care has been established [27]. NICE guidance recommends that any patient with suspected BPD should be referred to a psychiatrist for diagnosis and treatment planning [8]. Therefore, individuals in this cohort (psychiatrist-diagnosed BPD plus appropriate mood stabilizer treatment) are considered to fulfil International Statistical Classification of Diseases and Related Health Problems (ICD)-10 criteria for BPD.

In THIN, physicians use Read codes, a hierarchical coding system, to record information [28]. These codes include diagnoses made in primary and hospital care (which map onto ICD-10 codes), symptoms, examination findings, information from specialists, and test results [29]. In the UK, primary care physicians are responsible for drug prescriptions issued within the NHS, so this information is also complete and well recorded [30]. CKD, thyroid disease, T2DM, hypertension, CVD, and other chronic health condition diagnoses have been validated in THIN [23].

### Participants

Patients with a diagnosis of BPD were included if they had at least one 28-day prescription of lithium, valproate, olanzapine, or quetiapine after January 1, 1995 or after the date at which the medical records met quality assurance criteria for data entry (based on computer usage and mortality recording rates [31,32]). Patients were excluded if they were prescribed another study drug at the start of follow-up or in the month before this. Diagnosis of BPD could occur at any time in the patient record. For each outcome requiring hematological or biochemical confirmation for diagnosis (CKD, thyroid disease, hypercalcemia, hepatotoxicity), patients were excluded from the primary analysis if they did not receive a specific blood test for the outcome, to reduce surveillance bias. For the weight gain outcome, patients were excluded if they did not have a baseline or pre-treatment weight and at least one other weight measurement. For the outcome of hyperthyroidism, patients taking thyroxine were excluded, as this can result in thyroid-stimulating hormone (TSH) suppression [33]. Patients were also excluded if they had the outcome of interest at baseline (as we were interested in incident events). Therefore, each outcome has a different number of patients included.

### Exposure

Date of first prescription was taken as the start of exposure time. The end of the prescription was calculated from the amount prescribed and dosage instructions coded by the physician. Patients were considered to have a period of continuous prescribing if another prescription for the drug was issued within 3 mo of the calculated end date. If this did not occur, the date of stopping the study drug was the end date of the final prescription. Three mo was added to this end date to account for late development of the adverse event or delayed recording. Each patient could only contribute exposure time to one of the study drugs (the first they received) and did not re-enter the cohort if they restarted the drug after more than 3 mo. Patients could be prescribed other psychiatric medications but not combinations of the study drugs. If they commenced another study drug, their outcomes were censored in the analysis (to ensure the outcome could be assigned to a particular drug).

### Main Outcomes

All outcomes were defined by appropriate Read codes and/or lab results. Outcomes of interest were: CKD stage 3 or above (or an estimated glomerular filtration rate [eGFR] of <60 ml/min/1.73 m^2^), CKD stage 4 or above (or an eGFR <30 ml/min/1.73 m^2^) [34,35] (if eGFR was unavailable we calculated it from available creatinine blood tests using the CKD-EPI equation [36]), hypothyroidism (or a TSH of >10 mU/L), hyperthyroidism (or a TSH <0.1 mU/L) [33], hypercalcemia (adjusted calcium >2.65 mmol/L) [37], >7% and >15% weight gain from baseline [38], hypertension, T2DM (or HbA1c >48 mmol/mol) [39], CVD (defined as any ischemic heart disease [IHD], myocardial infarction [MI] or cerebrovascular event [CVE]), and hepatotoxicity (or alanine transaminase [ALT] >200 U/L, or aspartate aminotransferase [AST] >250 U/L) [40].

Patients were followed up until the earliest of (i) the first record of the adverse event of interest, (ii) the date of stopping the study drug plus 3 mo, (iii) the date of switching to another study drug, (iv) date of death or date of leaving the physician’s practice, or (v) December 31, 2013.

### Propensity Score Estimation Using Observed Pre-treatment Variables

A number of baseline patient characteristics were extracted from THIN. Physical and mental health conditions were considered present if referenced in patient notes and absent if they were not. If a patient had multiple entries of the same (or similar) codes, the start date of the condition was taken as the earliest date of entry.

A propensity score (PS) for each individual was estimated using variables defined a priori, based on existing research and clinical experience of factors influencing prescribing choice [3,41,42]. The PS is the conditional probability of receiving one study drug rather than another, given the variables included in the model [42,43]. Included variables were: sex, age at start of treatment with the study drug, year of entry to the cohort, ethnicity (grouped as White, Black, Asian, Mixed, other, with missing values coded as White [44]), IHD diagnosis before baseline, history of MI, history of CVE, hypertension, CKD at baseline (defined by Read code or blood test), history of hypo- or hyperthyroidism (defined by Read code or blood test), history of liver disease or hepatotoxicity (defined by Read code or blood test), T2DM (defined by Read code or blood test), epilepsy, alcohol use (grouped as none/low, moderate, high/dependent), history of illicit drug use, smoking status (grouped as never-smoker, ex-smoker, current smoker), body mass index (BMI) (grouped as healthy weight, overweight [BMI 25 to 30], obese [BMI over 30]), anxiety symptoms or diagnosis before baseline, depressive symptoms or diagnosis, sleep disturbance before baseline, treatment with one of the study drugs at or before baseline, and clustering by practice in which the treating physician was working. The PS was checked by comparison of covariate balance across treatments, within strata. The variables in the PS excluded the outcome variable for that particular analysis. Although PS estimation cannot remove all bias, it has been postulated to also reduce confounding from unmeasured covariates, because of their association with measured variables [45–47]. In this way, use of a PS aims to replicate a randomized experiment as closely as possible by obtaining treatment groups with similar covariate distributions [48].

### Statistical Analysis

Cox regression analyses were conducted, comparing the rates of adverse events in the four treatment groups. The proportional hazards model was tested formally with analysis of Schoenfeld residuals [49]. The PS was calculated using multinomial logistic regression, using drug treatment as the dependent variable and the covariates described as independent variables. The PS was then used as a linear term in a Cox regression analysis that also included age, calendar year, and clustering by practice [50]. In all cases, this model was shown to be superior to stratifying on PS using Akaike information criterion and Bayesian information criterion [51], and was a more efficient use of data than PS matching (because no patients were excluded). To account for the competing risk of each outcome with death, we plotted graphs of cumulative incidence function, adjusted for PS and age, following competing-risks regression [52,53]. We conducted sensitivity analyses in which individuals who did not receive blood tests or weight measurements were not dropped from the cohort, and in which individuals were assigned inverse probability weights (IPW) based on how likely they were to have blood test or weight records [54]. We used multiple demographic and clinical variables to predict missingness for the IPW model. All analyses were completed using Stata 14 [55].

## Results

For each outcome, 6,671 individuals with BPD diagnosis were potentially included in the analysis, 2,148 prescribed lithium, 1,670 prescribed valproate, 1,477 prescribed olanzapine, and 1,376 prescribed quetiapine (see S1 Text). The median duration of drug treatment was 1.48 y (interquartile range 0.64–3.43). The characteristics of the potentially included cohort are shown in Table 1. The number of individuals included for each outcome by treatment group is shown in S1 Table.

**Table 1:** Patient characteristics.

	lithium	valproate	olanzapine	quetiapine
Total, *n*	2,148	1,670	1,477	1,376
Female, *n* (%)	1,287 (59.92)	911 (54.55)	791 (53.55)	959 (69.69)
Age, median (IQR), years	46.28 (35.70–60.67)	42.31 (31.95–54.80)	41.01 (32.03–53.08)	38.08 (29.30–48.71)
Non-white ethnic background, *n* (%)	55 (2.56)	85 (5.09)	78 (5.28)	43 (3.13)
Duration of drug exposure, median (IQR), years	2.03 (0.77–4.86)	1.48 (0.65–3.35)	1.28 (0.59–3.29)	1.06 (0.56–2.26)
Primary care contacts per year, median (IQR)	11.14 (6.54–18.02)	12.51 (7.36–19.96)	11.94 (7.08–19.55)	14.61 (9.21–22.55)
**Health at baseline, *n* (%)**				
CVD history	124 (5.77)	121 (7.25)	68 (4.60)	53 (3.85)
≥CKD3 (or eGFR <60 ml/min/1.73m^2^)	52 (2.42)	40 (2.40)	27 (1.83)	32 (2.33)
Hypothyroidism (or TSH >10 mU/L)	183 (8.52)	105 (6.29)	60 (4.06)	61 (4.43)
Hyperthyroidism (or TSH <0.1 mU/L)	16 (0.74)	8 (0.48)	9 (0.61)	9 (0.65)
T2DM (or HbA1c >48 mmol/mol)	108 (5.03)	140 (8.38)	45 (3.05)	86 (6.25)
Hepatic impairment (or ALT >200 U/L or AST >250 U/L	34 (1.58)	41 (2.45)	37 (2.51)	19 (1.38)
Obesity (BMI >30)	896 (41.71)	716 (42.87)	509 (34.36)	609 (44.26)
Hypercalcemia (adjusted calcium >2.65 mmol/L)	10 (0.47)	4 (0.24)	2 (0.14)	3 (0.22)
Hypertension	184 (8.57)	173 (10.36)	103 (6.97)	130 (9.45)
Epilepsy	43 (2.00)	132 (7.90)	50 (3.39)	49 (3.56)
Previous anxiety problems	144 (6.70)	150 (8.98)	137 (9.28)	201 (14.61)
Moderate/heavy alcohol use	1,189 (55.35)	899 (53.83)	791 (53.55)	708 (51.45)
Current smoker	711 (33.10)	652 (39.04)	632 (42.79)	567 (41.21)
**Bipolar disorder characteristics at baseline, *n* (%)**				
Previous depressive episode	1,238 (57.64)	990 (59.28)	915 (61.95)	1,015 (73.76)
Previous record of taking study drug	1,731 (80.59)	1,157 (69.28)	886 (59.99)	847 (61.56)

CVD, cardiovascular disease; CKD, chronic kidney disease; eGFR, estimated glomerular filtration rate; TSH, thyroid stimulating hormone; T2DM, type 2 diabetes mellitus; ALT, alanine transaminase; AST, aspartate aminotransferase; BMI, body mass index

**Fig 1:**
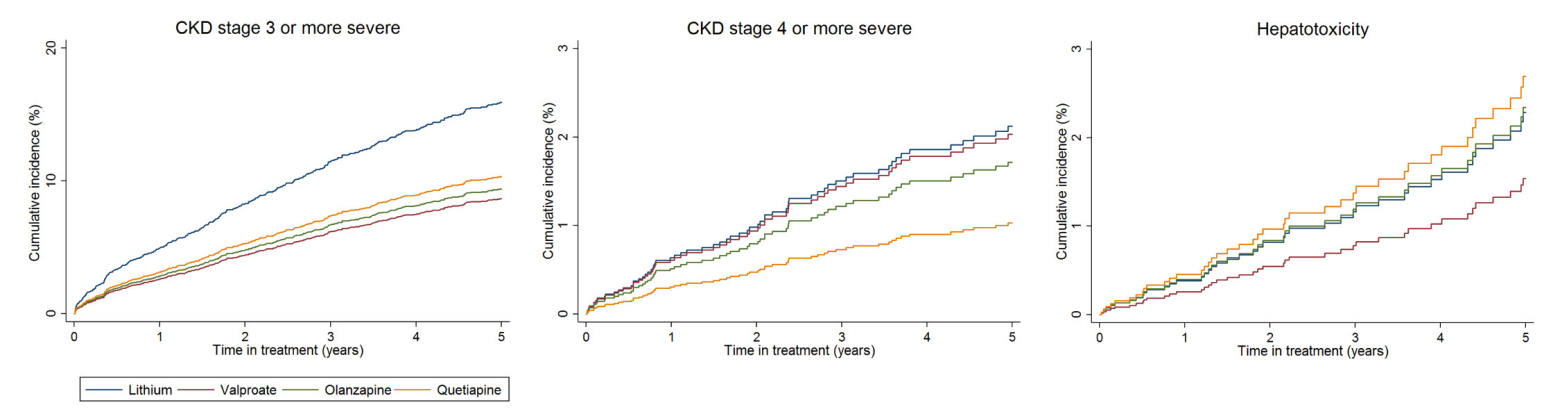
Cumulative incidence estimates of adverse renal and hepatic event rates. From PS and age-adjusted competing-risks regression. Note differences in scale of *y*-axis for each plot.

In unadjusted analysis and after adjustment for PS, age, calendar year, and clustering by practice in which the primary care physician worked, rates of CKD stage 3 or above in individuals prescribed valproate (HR 0.56; 95% CI 0.45–0.69; *p* < 0.001), olanzapine (HR 0.57; 95% CI 0.45–0.71; *p* < 0.001), or quetiapine (HR 0.62; 95% CI 0.47–0.80; *p* < 0.001) were reduced compared to lithium (Table 2, Fig 1).

**Table 2:** Adverse effects during maintenance treatment.

	Lithium	Valproate	Olanzapine	Quetiapine
**≥CKD stage 3** (*n* = 4,560)				
Events, *n*	489	130	121	71
PYAR (100s)	51.97	29.85	25.64	16.89
Rate, per 100 PYAR (95%CI)	9.41 (8.61–10.28)	4.35 (3.67–5.17)	4.72 (3.95–5.64)	4.20 (3.33–5.31)
Unadjusted HR (95%CI)	1 (reference)	0.46 (0.38–0.55)	0.50 (0.41–0.61)	0.43 (0.33–0.55)
PS Adjusted HR (95%CI)	1 (reference)	0.56 (0.45–0.69)	0.57 (0.45–0.71)	0.62 (0.47–0.80)
*p*-value		<0.001	<0.001	<0.001
**≥CKD stage 4** (*n* = 4,817)				
Events, *n*	91	34	20	12
PYAR (100s)	63.48	32.75	27.77	18.35
Rate, per 100 PYAR (95%CI)	1.43 (1.17–1.76)	1.04 (0.74–1.45)	0.72 (0.46–1.12)	0.65 (0.37–1.15)
Unadjusted HR (95%CI)	1 (reference)	0.75 (0.51–1.13)	0.52 (0.32–0.85)	0.47 (0.25–0.87)
PS Adjusted HR (95%CI)	1 (reference)	0.94 (0.59–1.50)	0.65 (0.37–1.12)	0.67 (0.33–1.37)
*p*-value		0.806	0.127	0.273
**Hypothyroidism** (*n* = 4,093)				
Events, *n*	183	61	41	33
PYAR (100s)	59.23	27.78	23.79	15.59
Rate, per 100 PYAR (95%CI)	3.09 (2.67–3.57)	2.20 (1.71–2.82)	1.72 (1.27–2.34)	2.12 (1.50–2.98)
Unadjusted HR (95%CI)	1 (reference)	0.69 (0.51–0.94)	0.54 (0.38–0.77)	0.62 (0.42–0.90)
PS Adjusted HR (95%CI)	1 (reference)	0.60 (0.40–0.89)	0.48 (0.29–0.77)	0.63 (0.38–1.05)
*p*-value		0.012	0.003	0.074
**Hyperthyroidism** (*n* = 3,704)				
Events, *n*	41	5	6	6
PYAR (100s)	52.49	25.81	22.62	14.65
Rate, per 100 PYAR (95%CI)	0.78 (0.58–1.06)	0.19 (0.08–0.47)	0.27 (0.12–0.59)	0.41 (0.18–0.91)
Unadjusted HR (95%CI)	1 (reference)	0.24 (0.09–0.61)	0.33 (0.14–0.78)	0.48 (0.20–1.17)
PS Adjusted HR (95%CI)	1 (reference)	0.24 (0.09–0.61)	0.31 (0.13–0.73)	0.45 (0.18–1.18)
*p*-value		0.003	0.007	0.096
**Hypercalcemia** (*n* = 2,094)				
Events, *n*	55	6	6	3
PYAR (100s)	36.29	17.1	13.44	8.99
Rate, per 100 PYAR (95%CI)	1.52 (1.16–1.97)	0.35 (0.16–0.78)	0.45 (0.20–0.99)	0.33 (0.11–1.03)
Unadjusted HR (95%CI)	1 (reference)	0.24 (0.10–0.56)	0.31 (0.14–0.68)	0.24 (0.07–0.76)
PS Adjusted HR (95%CI)	1 (reference)	0.25 (0.10–0.60)	0.32 (0.14–0.76)	0.23 (0.07–0.73)
*p*-value		0.002	0.008	0.013
**T2DM** (*n* = 6,292)				
Events, *n*	150	86	88	51
PYAR (100s)	69.11	36.52	34.01	21.28
Rate, per 100 PYAR (95%CI)	2.17 (1.85–2.55)	2.35 (1.91–2.91)	2.59 (2.10–3.19)	2.40 (1.82–3.15)
Unadjusted HR (95%CI)	1 (reference)	1.21 (0.94–1.55)	1.31 (0.99–1.74)	1.32 (0.95–1.82)
PS Adjusted HR (95%CI)	1 (reference)	1.08 (0.83–1.42)	1.20 (0.89–1.61)	0.94 (0.65–1.35)
*p*-value		0.586	0.230	0.752
**CVD** (*n* = 6,305)				
Events, *n*	94	32	26	21
PYAR (100s)	69.49	37.56	33.41	22.17
Rate, per 100 PYAR (95%CI)	1.35 (1.11–1.66)	0.85 (0.60–1.20)	0.78 (0.53–1.14)	0.95 (0.62–1.45)
Unadjusted HR (95%CI)	1 (reference)	0.67 (0.44–1.04)	0.61 (0.38–0.98)	0.79 (0.47–1.35)
PS Adjusted HR (95%CI)	1 (reference)	0.91 (0.59–1.41)	0.85 (0.53–1.36)	1.11 (0.63–1.96)
*p*-value		0.684	0.509	0.732
**>7% weight gain** (*n* = 4,458)				
Events, *n*	467	410	396	299
PYAR (100s)	63.20	33.28	30.56	19.05
Rate, per 100 PYAR (95%CI)	7.39 (6.75–8.09)	12.32 (11.18–13.57)	12.96 (11.74–14.30)	15.70 (14.02–17.58)
Unadjusted HR (95%CI)	1 (reference)	1.90 (1.64–2.20)	1.99 (1.72–2.30)	2.72 (2.33–3.16)
PS Adjusted HR (95%CI)	1 (reference)	1.37 (1.17–1.61)	1.43 (1.23–1.67)	1.37 (1.16–1.62)
*p*-value		<0.001	<0.001	<0.001
**>15% weight gain** (*n* = 4,458)				
Events, *n*	179	182	189	130
PYAR (100s)	63.92	34.27	31.24	19.30
Rate, per 100 PYAR (95%CI)	2.80 (2.42–3.24)	5.31 (4.59–6.14)	6.05 (5.25–6.98)	6.74 (5.67–8.00)
Unadjusted HR (95%CI)	1 (reference)	2.29 (1.87–2.82)	2.57 (2.06–3.22)	3.41 (2.61–4.44)
PS Adjusted HR (95%CI)	1 (reference)	1.62 (1.31–2.01)	1.84 (1.47–2.30)	1.67 (1.24–2.20)
*p*-value		<0.001	<0.001	<0.001
**Hypertension** (*n* = 6,081)				
Events, *n*	174	85	89	33
PYAR (100s)	64.25	35.66	32.30	20.65
Rate, per 100 PYAR (95%CI)	2.71 (2.33–3.14)	2.38 (1.93–2.95)	2.76 (2.24–3.39)	1.60 (1.14–2.24)
Unadjusted HR (95%CI)	1 (reference)	0.98 (0.75–1.26)	1.11 (0.84–1.46)	0.75 (0.50–1.12)
PS Adjusted HR (95%CI)	1 (reference)	1.19 (0.90–1.58)	1.41 (1.06–1.87)	0.89 (0.59–1.34)
*p*-value		0.274	0.017	0.590
**Hepatotoxicity** (*n* = 3,352)				
Events, *n*	20	10	14	13
PYAR (100s)	50.13	25.95	20.39	12.53
Rate, per 100 PYAR (95%CI)	0.40 (0.26–0.62)	0.39 (0.21–0.72)	0.69 (0.41–1.16)	1.04 (0.60–1.79)
Unadjusted HR (95%CI)	1 (reference)	0.96 (0.45–2.07)	1.71 (0.86–3.40)	2.59 (1.26–5.32)
PS Adjusted HR (95%CI)	1 (reference)	0.65 (0.30–1.39)	1.23 (0.63–2.42)	1.21 (0.54–2.74)
*p*-value		0.274	0.558	0.658

CKD, chronic kidney disease; T2DM, type 2 diabetes mellitus; CVD, cardiovascular disease; PYAR, person years at risk; HR, hazard ratio; PS, propensity score. Unadjusted hazard ratio accounts for clustering by primary care practice, adjusted hazard ratio is adjusted for propensity score age group and calendar period time varying variables and clustering by primary care practice. P-values for PS adjusted HR.

**Fig 2:**
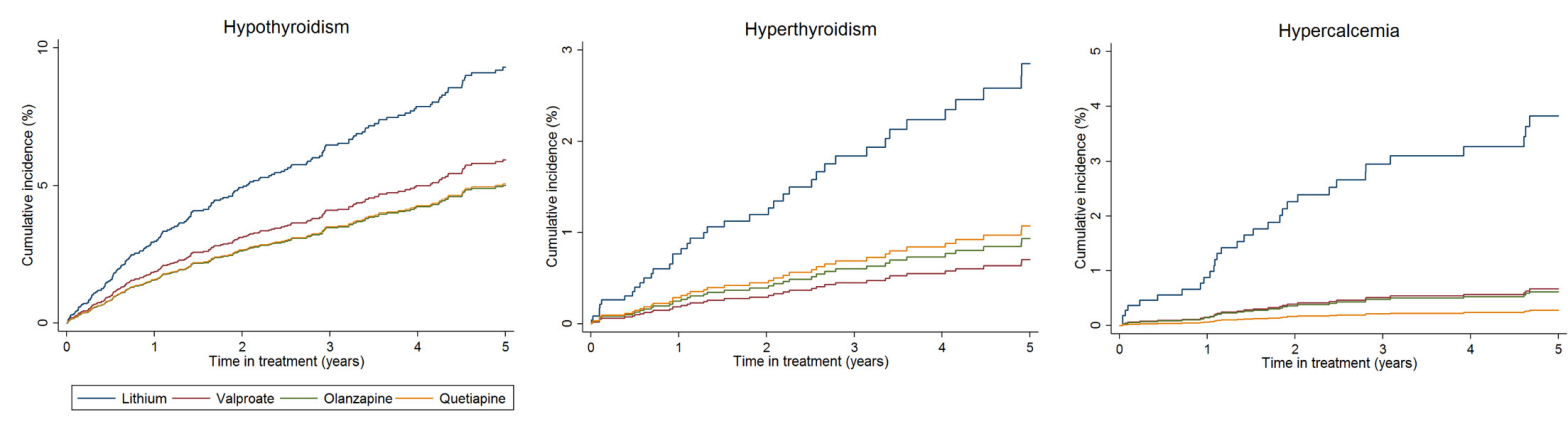
Cumulative incidence estimates of adverse endocrine event rates. From PS and age adjusted competing-risks regression. Note differences in scale of *y*-axis for each plot.

Compared to lithium, rates of hypothyroidism were reduced in those prescribed valproate (HR 0.60; 95% CI 0.40–0.89; *p* = 0.012) or olanzapine (HR 0.48; 95% CI 0.29–0.77; *p* = 0.003), but not quetiapine (HR 0.63; 95% CI 0.38–1.05; *p* = 0.074) after adjustment. Rates of hyperthyroidism were lower in those prescribed valproate (HR 0.24; 95% CI 0.09–0.61; 0.003) and olanzapine (HR 0.31; 95% CI 0.13–0.73; 0.007), but not quetiapine (HR 0.45; 95% CI 0.18–1.18; 0.096), compared to lithium. Hypercalcemia was less common in those prescribed valproate (HR 0.25; 95% CI 0.10–0.60; *p* = 0.002), olanzapine (HR 0.32; 95% CI 0.14–0.76; *p* = 0.008), or quetiapine (HR 0.23; 95% CI 0.07–0.73; *p* = 0.013) compared to lithium (Table 2, Fig 2).

**Fig 3:**
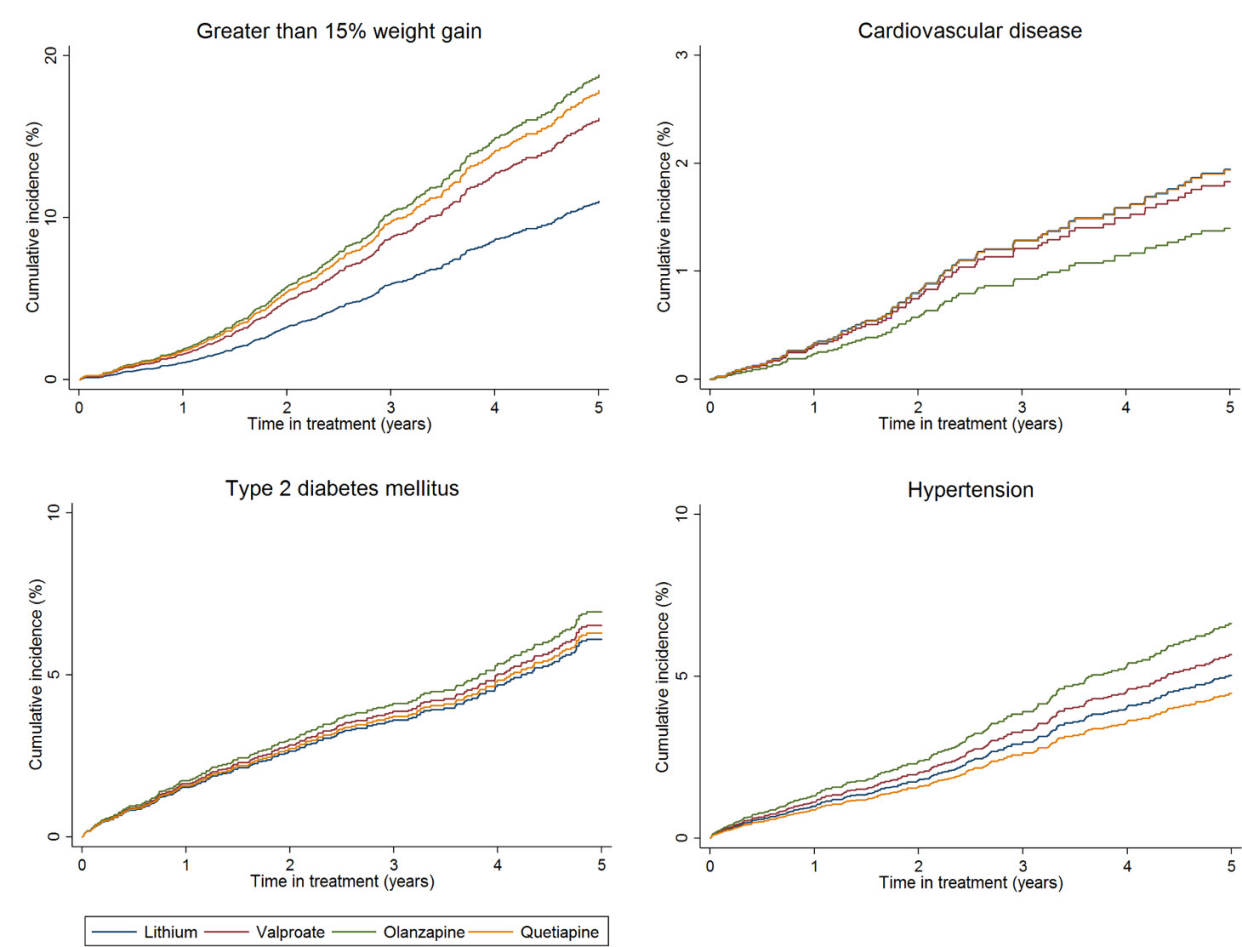
Cumulative incidence estimates of adverse metabolic event rates. From PS and age adjusted competing-risks regression. Note differences in scale of *y*-axis for each plot.

After adjustment, rates of weight gain were higher with valproate, olanzapine, and quetiapine than lithium (>15% weight gain: valproate HR 1.62; 95% CI 1.31–2.01; *p* < 0.001; olanzapine HR 1.84; 95% CI 1.47–2.30; *p* < 0.001; quetiapine HR 1.67; 95% CI 1.24–2.20; *p* < 0.001). Rates of hypertension were higher with olanzapine (HR 1.41; 95% CI 1.06–1.87; *p* = 0.017) than lithium (Table 2, Fig 3). We found no significant difference in rates of CKD stage 4 or above, T2DM, cardiovascular disease, or hepatotoxicity between groups (Table 2). The median number of eGFR/creatinine and TSH blood tests per year in treatment was higher in those taking lithium than the other drugs (see S2 Table). Weight measurement and blood tests for adjusted calcium and ALT/AST were less frequent in patients prescribed lithium (see S2 Table). For outcomes in which patients had been excluded because of missing tests (CKD, hypo- and hyperthyroidism, hypercalcemia, weight gain, and hepatotoxicity), sensitivity analyses including all patients resulted in reduced incident rate estimates compared to the primary analyses, but had little effect on HRs (see S3 Table). Sensitivity analyses using IPW suggest results from the primary analyses are robust (see S3 Table). From Schoenfeld residuals, there was no evidence against the assumption of proportional hazards for any outcome.

## Discussion

In a large dataset of nearly 7,000 individuals treated for BPD with lithium, valproate, olanzapine, or quetiapine, with follow-up times of up to 17 y, we found differential rates of a number of adverse events. Those prescribed lithium were more likely to have a decline in renal function and develop hypo- or hyperthyroidism and hypercalcemia. However, they were less likely to gain significant weight. Individuals prescribed olanzapine had the highest rate of weight gain and new onset hypertension. We did not find any statistically significant differences in the rate of new T2DM, cardiovascular disease, or hepatotoxicity across drug treatment groups.

Severe CKD (stage 4 or above) was uncommon in the cohort (approximately 1 in 100 person years at risk), and we did not find differences by drug treatment, but less severe CKD (stage 3 or above) occurred most frequently in patients prescribed lithium. Whilst many of these patients (i.e., those with CKD stage 3) would not progress to a clinically relevant decline in renal function, a number of them would be at increased risk of doing so. It remains unclear if this result is due to (1) lack of power to determine a true difference in rates of severe CKD, (2) surveillance bias due to increased monitoring of renal function in those taking lithium, which would lead to apparent increased rates of asymptomatic CKD stage 3, or (3) lithium treatment truly increasing the risk of reduced renal function without increasing severe CKD risk. Previous studies have found similar results and have not been able to account for this potential bias [12–14,56]. Clos et al. found no decline in eGFR in individuals taking lithium, using a similar active comparator design, but were also limited by potential ascertainment bias [57].

Rates of both hypothyroidism and hyperthyroidism were increased in individuals prescribed lithium compared to valproate and olanzapine (but not quetiapine). Increased hypothyroidism has been shown previously [11,58], but literature on the association between lithium and hyperthyroidism is inconsistent [13], and lithium-induced hyperthyroidism is considered rare [59]. Monitoring thyroid dysfunction in BPD is vital because of evidence that abnormalities are associated with longer time to remission and more symptoms during the maintenance period [60]. It is possible that thyroid function normalises on cessation of lithium, but only one study has investigated this [61]. Hypercalcemia is also recognised to be associated with lithium prescribing [11,13,62,63]. Calcium monitoring in patients prescribed lithium was rare in our representative sample of primary care (37% had one or more calcium blood test result), despite it being recommended in the 2006 NICE guidance [8].

The rate of individuals gaining more than 7%, and more than 15% of their baseline weight, was greater in those prescribed olanzapine, quetiapine, or valproate than those prescribed lithium. This degree of weight gain represents a significant risk factor for a number of adverse physical health outcomes, including CVD and T2DM [38]. We may not have captured increased rates of CVD or T2DM because of the relatively brief median follow-up time, in relation to the time taken to develop these diseases. Olanzapine had the highest adjusted rate of greater than 15% weight gain compared to lithium, and the highest rate of new onset hypertension. This has been shown previously in comparisons of antipsychotic drugs [64] and in trials of olanzapine versus lithium or valproate [65].

Hepatotoxicity was rare in the cohort and, before PS adjustment rates, appeared to be elevated in the quetiapine group, compared to lithium. This association has been identified previously [20]. After adjustment, there was no evidence of between-group differences.

### Strengths and Limitations

The major strength of this study, beyond size and length of follow-up, is the direct comparison between BPD maintenance mood stabilizer treatment options for a number of adverse effects. The use of electronic health records also means it is possible to adjust for a number of demographic and physical health characteristics that may have influenced the clinician’s decision to treat with a particular medication or potentially confound the relationship between treatment and adverse outcome. Despite including numerous variables in the PS, it is possible that residual confounding remained, especially as those prescribed lithium were older and were more likely to have taken the drug previously, perhaps reflecting a more chronic illness course. It may be that important patient or clinician features were not captured by the score, and despite the balance of observed covariates, we cannot confirm balance of unobserved covariates [66,67]. We were also unable to consider dosage differences across the different treatment groups in this analysis. Periods of lithium toxicity may be particularly important with regards to developing renal failure, and we were unable to capture this information from the available data. Missing data can be a problem in studies utilising electronic patient records, especially as there may be a clinical reason why information is missing. Because of the way outcomes were defined, T2DM, cardiovascular disease, and diagnoses of hypertension had no missing data, and no covariates in the PS had missing values.

Patients prescribed lithium had no more physician contacts than those taking other mood stabilizer medication. In individuals that ever received tests during treatment exposure, testing frequency was similar in all study drugs for adjusted calcium, liver function, and weight (see S2 Table). Frequency of testing renal and thyroid function was higher in those taking lithium, which reflects the guidance for monitoring [8]. Patients prescribed lithium were also more likely to have at least one renal function, thyroid function, calcium, or liver function test compared to patients taking other drugs. This is likely to be due to both drug-related indications for monitoring and the longer drug exposure seen in those taking lithium. IPW sensitivity analysis to account for this difference did not alter our conclusions (see S3 Table). In the primary analysis, the likely effect of this differential missingness would be to reduce the hazard ratios for lithium compared to the other drugs, relative to their true values, as blood tests in the non-lithium group are more likely to be related to clinical symptoms than monitoring guidance (for instance, this is likely to represent an underestimation of the true hypercalcemia hazard ratio for lithium versus other drugs). The median number of weight measurements was similar in each group, suggesting detection of weight gain was not related to differential monitoring. The sensitivity analyses including individuals irrespective of blood tests produced similar adjusted hazard ratios as the primary analyses for each outcome, but often with reduced incidence of the outcome in each treatment group (see S3 Table). These analyses may more accurately reflect testing occurring because of clinical indication.

## Conclusions

Lithium remains an important treatment option for individuals with BPD. However, there is clear evidence that its use is associated with a number of adverse events. These risks need to be offset with the potentially superior effectiveness and anti-suicidal benefits of the drug compared to other treatment options [5,68]. It is also true that other recommended maintenance treatments can have serious side effects, often related to weight gain, and are not suitable for use in certain patient groups (such as the contraindication of valproate in women of childbearing potential [3]).

Assiduous monitoring of patients prescribed lithium should ameliorate some risk associated with effects on renal physiology and endocrine systems. Given the need to balance an array of risks and benefits, an individualised and collaborative approach to treatment choice is likely to be most appropriate. To achieve this, further research identifying patient characteristics that are risk factors for specific side effects and an understanding of the risks and benefits of stopping treatment in those who experience adverse effects is necessary.

## Supporting Information

S1 Table Patients included for each outcome, *n* (%). (DOCX)

S2 Table Median number (and interquartile range) of tests per year of drug exposure in patients included in analyses. (DOCX)

S3 Table Sensitivity analyses to account for missing blood tests by (1) including all individuals and (2) performing inverse probability weighting. (DOCX)

S1 Text Patient selection. (DOCX)

S2 Text STROBE statement. (DOCX)

S3 Text Prospective analysis plan. (DOCX)

## Abbreviations

ALT: alanine transaminase
AST: aspartate aminotransferase
BMI: body mass index
BPD: bipolar disorder
CKD: chronic kidney disease
CVD: cardiovascular disease
CVE: cerebrovascular event
eGFR: estimated glomerular filtration rate
ICD-10: International Statistical Classification of Diseases and Related Health Problems
IHD: ischemic heart disease
IPW: inverse probability weights
MI: myocardial infarction
NHS: National Health Service
NICE: National Institute for Health and Care Excellence
PS: propensity score
T2DM: type 2 diabetes mellitus
THIN: The Health Improvement Network
TSH: thyroid-stimulating hormone
