# Supplementary material for: Adverse Renal, Endocrine, Hepatic, and Metabolic Events during Maintenance Mood Stabilizer Treatment for Bipolar Disorder: A Population-Based Cohort Study
Source: PLoS Med. 2016 Aug 2;13(8):e1002058. doi: 10.1371/journal.pmed.1002058 (PMC4970809; doi:10.1371/journal.pmed.1002058)
Supplement: S1 Table — (DOCX) [file pmed.1002058.s001.docx]

**S1 Table. Patients included for each outcome, N (%)**

|  | **Lithium** | **Valproate** | **Olanzapine** | **Quetiapine** |
| --- | --- | --- | --- | --- |
| Total potentially included, N | 2148 | 1670 | 1477 | 1376 |
| **≥CKD stage 3** | 1541 (71.74) | 1116 (66.83) | 964 (65.27) | 939 (68.24) |
| **≥CKD stage 4** | 1642 (76.44) | 1176 (70.42) | 1016 (68.79) | 983 (71.44) |
| **Hypothyroidism** | 1620 (74.95) | 916 (54.85) | 832 (56.33) | 735 (53.42) |
| **Hyperthyroidism** | 1398 (65.08) | 844 (50.54) | 775 (52.47) | 687 (49.93) |
| **Hypercalcemia** | 785 (36.55) | 513 (30.72) | 408 (27.62) | 388 (28.20) |
| **T2DM** | 2040 (94.97) | 1530 (91.62) | 1432 (96.95) | 1290 (93.75) |
| **CVD** | 2024 (94.23) | 1549 (92.75) | 1409 (95.40) | 1323 (96.15) |
| **Weight gain** | 1426 (66.39) | 1116 (66.83) | 1004 (67.98) | 912 (66.28) |
| **Hypertension** | 1964 (91.43) | 1497 (89.64) | 1374 (93.03) | 1246 (90.55) |
| **Hepatotoxicity** | 1171 (54.52) | 852 (51.02) | 718 (48.61) | 611 (44.40) |

CKD chronic kidney disease; T2DM type 2 diabetes mellitus; CVD cardiovascular disease
