# Supplementary material for: Adverse Renal, Endocrine, Hepatic, and Metabolic Events during Maintenance Mood Stabilizer Treatment for Bipolar Disorder: A Population-Based Cohort Study
Source: PLoS Med. 2016 Aug 2;13(8):e1002058. doi: 10.1371/journal.pmed.1002058 (PMC4970809; doi:10.1371/journal.pmed.1002058)
Supplement: S2 Table — (DOCX) [file pmed.1002058.s002.docx]

**S2 Table. Median number (and interquartile range) of tests per year of drug exposure in patients included in analyses**

|  | **Lithium** | **Valproate** | **Olanzapine** | **Quetiapine** |
| --- | --- | --- | --- | --- |
| eGFR or creatinine | 1.84 (1.04-3.03) | 1.10 (0.62-1.88) | 1.00 (0.56-1.78) | 1.20 (0.69-2.01) |
| TSH | 1.64 (1.05-2.47) | 0.85 (0.49-1.46) | 0.80 (0.45-1.50) | 1.04 (0.62-1.70) |
| Adjusted calcium | 0.91 (0.38-2.18) | 0.90 (0.41-2.12) | 1.10 (0.43-2.99) | 1.37 (0.62-2.99) |
| ALT or AST | 0.75 (0.36-1.51) | 0.81 (0.40-1.57) | 0.73 (0.37-1.40) | 0.94 (0.49-1.70) |
| Weight | 0.98 (0.53-1.73) | 1.19 (0.73-2.07) | 1.20 (0.63-2.01) | 1.44 (0.89-2.44) |

eGFR estimated glomerular filtration rate; TSH thyroid stimulating hormone; ALT alanine transaminase; AST aspartate aminotransferase. Type 2 diabetes mellitus, cardiovascular disease and hypertension were not defined by tests
