# Supplementary material for: Adverse Renal, Endocrine, Hepatic, and Metabolic Events during Maintenance Mood Stabilizer Treatment for Bipolar Disorder: A Population-Based Cohort Study
Source: PLoS Med. 2016 Aug 2;13(8):e1002058. doi: 10.1371/journal.pmed.1002058 (PMC4970809; doi:10.1371/journal.pmed.1002058)
Supplement: S3 Table — (DOCX) [file pmed.1002058.s003.docx]

**S3 Table. Sensitivity analyses to account for missing blood tests by 1) including all individuals and 2) performing inverse probability weighting**

|  | **Lithium** | **Valproate** | **Olanzapine** | **Quetiapine** |
| --- | --- | --- | --- | --- |
| **≥CKD stage 3** (N=6520) |  |  |  |  |
| Events, N | 489 | 130 | 121 | 71 |
| PYAR (100s) | 60.65 | 36.62 | 32.56 | 21.33 |
| Rate, per 100 PYAR  (95% CI) | 8.06  (7.38-8.81) | 3.55  (2.99-4.22) | 3.72  (3.11-4.44) | 3.33  (2.64-4.20) |
| Unadjusted HR  (95%CI)  P-value | 1 [reference] | 0.44  (0.37-0.54)  <0.001 | 0.46  (0.38-0.57)  <0.001 | 0.41  (0.32-0.53)  <0.001 |
| PS Adjusted HR  (95%CI)  P-value | 1 [reference] | 0.54  (0.44-0.67)  <0.001 | 0.55  (0.44-0.70)  <0.001 | 0.55  (0.42-0.72)  <0.001 |
| IPW PS Adjusted HR  (95%CI)  P-value | 1 [reference] | 0.63  (0.51-0.78)  <0.001 | 0.67  (0.53-0.83)  <0.001 | 0.70  (0.52-0.93)  0.016 |
| **≥CKD stage 4** (N=6600) |  |  |  |  |
| Events, N | 91 | 34 | 20 | 12 |
| PYAR (100s) | 72.26 | 39.58 | 34.70 | 22.81 |
| Rate, per 100 PYAR  (95% CI) | 1.25  (1.03-1.56) | 0.86  (0.61-1.20) | 0.58  (0.37-0.89) | 0.53  (0.30-0.93) |
| Unadjusted HR  (95%CI)  P-value | 1 [reference] | 0.73  (0.49-1.08)  0.118 | 0.49  (0.30-0.79)  0.003 | 0.45  (0.24-0.83)  0.012 |
| PS Adjusted HR  (95%CI)  P-value | 1 [reference] | 0.90  (0.57-1.43)  0.666 | 0.61  (0.36-1.05)  0.070 | 0.58  (0.28-1.18)  0.138 |
| IPW PS Adjusted HR  (95%CI)  P-value | 1 [reference] | 1.04  (0.64-1.68)  0.883 | 0.70  (0.40-1.21)  0.208 | 0.77  (0.36-1.64)  0.509 |
| **Hypothyroidism** (N=6262) |  |  |  |  |
| Events, N | 183 | 61 | 41 | 33 |
| PYAR (100s) | 63.69 | 37.20 | 33.50 | 22.03 |
| Rate, per 100 PYAR  (95% CI) | 2.87  (2.49-3.32) | 1.64  (1.28-2.10) | 1.22  (0.90-1.66) | 1.50  (1.06-2.11) |
| Unadjusted HR  (95%CI)  P-value | 1 [reference] | 0.56  (0.41-0.76)  <0.001 | 0.41  (0.29-0.59)  <0.001 | 0.48  (0.33-0.70)  <0.001 |
| PS Adjusted HR  (95%CI)  P-value | 1 [reference] | 0.59  (0.42-0.84)  0.003 | 0.43  (0.29-0.63)  <0.001 | 0.47  (0.31-0.73)  <0.001 |
| IPW PS Adjusted HR  (95%CI)  P-value | 1 [reference] | 0.54  (0.38-0.78)  <0.001 | 0.42  (0.28-0.63)  <0.001 | 0.49  (0.32-0.78)  0.002 |
| **Hyperthyroidism** (N=6220) |  |  |  |  |
| Events, N | 41 | 5 | 6 | 6 |
| PYAR (100s) | 56.79 | 35.27 | 32.41 | 21.11 |
| Rate, per 100 PYAR  (95% CI) | 0.72  (0.53-0.98) | 0.14  (0.06-0.34) | 0.19  (0.08-0.41) | 0.28  (0.13-0.62) |
| Unadjusted HR  (95%CI)  P-value | 1 [reference] | 0.19  (0.07-0.49)  <0.001 | 0.25  (0.11-0.59)  0.001 | 0.37  (0.15-0.91)  0.030 |
| PS Adjusted HR  (95%CI)  P-value | 1 [reference] | 0.19  (0.07-0.51)  <0.001 | 0.25  (0.11-0.59)  0.001 | 0.34  (0.13-0.90)  0.029 |
| IPW PS Adjusted HR  (95%CI)  P-value | 1 [reference] | 0.20  (0.08-0.53)  <0.001 | 0.28  (0.12-0.67)  0.004 | 0.34  (0.14-0.99)  0.030 |
| **Hypercalcemia** (N=6652) |  |  |  |  |
| Events, N | 55 | 6 | 6 | 3 |
| PYAR (100s) | 72.59 | 40.23 | 35.03 | 23.12 |
| Rate, per 100 PYAR  (95% CI) | 0.76  (0.58-0.99) | 0.15  (0.07-0.33) | 0.17  (0.08-0.38) | 0.13  (0.04-0.40) |
| Unadjusted HR  (95%CI)  P-value | 1 [reference] | 0.22  (0.09-0.51)  <0.001 | 0.24  (0.11-0.54)  <0.001 | 0.20  (0.06-0.64)  0.008 |
| PS Adjusted HR  (95%CI)  P-value | 1 [reference] | 0.23  (0.09-0.56)  <0.001 | 0.28  (0.12-0.66)  0.003 | 0.21  (0.06-0.68)  0.012 |
| IPW PS Adjusted HR  (95%CI)  P-value | 1 [reference] | 0.22  (0.09-0.57)  <0.001 | 0.24  (0.10-0.55)  0.001 | 0.22  (0.06-0.76)  0.019 |
| **>7% weight gain** (N=6671) |  |  |  |  |
| Events, N | 467 | 410 | 396 | 299 |
| PYAR (100s) | 73.36 | 39.54 | 35.96 | 23.09 |
| Rate, per 100 PYAR  (95% CI) | 6.35 (5.80-6.96) | 10.37  (9.41-11.42) | 11.01  (9.98-12.15) | 12.90  (11.52-14.46) |
| Unadjusted HR  (95%CI)  P-value | 1 [reference] | 1.91  (1.65-2.21)  <0.001 | 2.01  (1.74-2.33)  <0.001 | 2.77  (2.37-3.22)  <0.001 |
| PS Adjusted HR  (95%CI)  P-value | 1 [reference] | 1.37  (1.17-1.61)  <0.001 | 1.46  (1.25-1.71)  <0.001 | 1.28  (1.09-1.52)  0.004 |
| IPW PS Adjusted HR  (95%CI)  P-value | 1 [reference] | 1.23  (1.08-1.41)  0.002 | 1.30  (1.14-1.47)  <0.001 | 1.52  (1.33-1.75)  <0.001 |
| **>15% weight gain** (N=6671) |  |  |  |  |
| Events, N | 179 | 182 | 189 | 130 |
| PYAR (100s) | 73.95 | 40.53 | 36.65 | 23.35 |
| Rate, per 100 PYAR  (95% CI) | 2.43  (2.10-2.82) | 4.49  (3.88-5.19) | 5.16  (4.47-5.95) | 5.57  (4.69-6.61) |
| Unadjusted HR  (95%CI)  P-value | 1 [reference] | 2.28  (1.85-2.81)  <0.001 | 2.57  (2.05-3.22)  <0.001 | 3.44  (2.63-4.47)  <0.001 |
| PS Adjusted HR  (95%CI)  P-value | 1 [reference] | 1.63  (1.31-2.02)  <0.001 | 1.86  (1.49-2.33)  <0.001 | 1.54  (1.15-2.05)  0.003 |
| IPW PS Adjusted HR  (95%CI)  P-value | 1 [reference] | 1.35  (1.15-1.58)  <0.001 | 1.44  (1.23-1.69)  <0.001 | 1.62  (1.32-1.98)  <0.001 |
| **Hepatotoxicity** (N=6540) |  |  |  |  |
| Events, N | 20 | 10 | 14 | 13 |
| PYAR (100s) | 72.08 | 39.57 | 34.13 | 22.86 |
| Rate, per 100 PYAR  (95% CI) | 0.28  (0.18-0.43) | 0.25  (0.14-0.47) | 0.41  (0.24-0.69) | 0.57  (0.33-0.98) |
| Unadjusted HR  (95%CI)  P-value | 1 [reference] | 0.94  (0.44-2.02)  0.883 | 1.53  (0.77-3.06)  0.229 | 2.22  (1.07-4.57)  0.031 |
| PS Adjusted HR  (95%CI)  P-value | 1 [reference] | 0.63  (0.30-1.34)  0.228 | 1.05  (0.53-2.08)  0.897 | 0.98  (0.45-2.16)  0.963 |
| IPW PS Adjusted HR  (95%CI)  P-value | 1 [reference] | 0.58  (0.27-1.26)  0.166 | 0.97  (0.48-1.94)  0.938 | 1.12  (0.48-2.59)  0.804 |

CKD chronic kidney disease; PYAR person-years at risk; HR hazard ratio; PS propensity score IPW inverse probability weighted; outcomes for cardiovascular disease, type 2 diabetes mellitus and hypertension had no missing data. N for each outcome varies because of potential diagnoses pre-baseline. Unadjusted HR accounts for clustering by primary care practice, PS adjusted HR is adjusted for propensity score, age group and calendar period time varying variables and clustering by primary care practice, IPW PS adjusted HR accounts for probability of being a complete record, adjusted for propensity score, age group and calendar period time varying variables and clustering by primary care practice.
