# Supplementary figures and images for: Adverse Renal, Endocrine, Hepatic, and Metabolic Events during Maintenance Mood Stabilizer Treatment for Bipolar Disorder: A Population-Based Cohort Study
Source: PLoS Med. 2016 Aug 2;13(8):e1002058. doi: 10.1371/journal.pmed.1002058 (PMC4970809; doi:10.1371/journal.pmed.1002058)

**S1 Text. Patient selection**

**
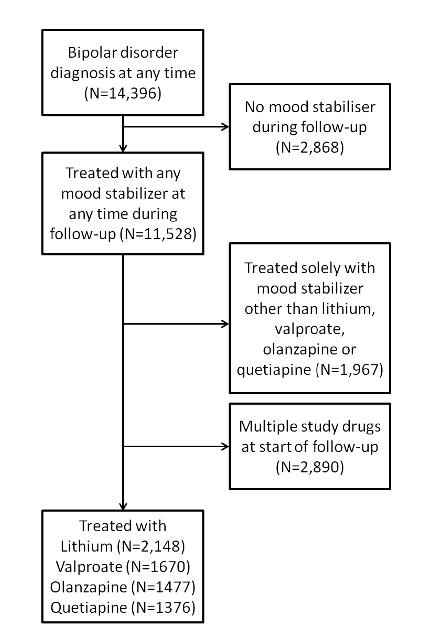
**

Supplement: S1 Text — (DOCX) [file pmed.1002058.s004.docx]
