# Supplementary material for: Adverse Renal, Endocrine, Hepatic, and Metabolic Events during Maintenance Mood Stabilizer Treatment for Bipolar Disorder: A Population-Based Cohort Study
Source: PLoS Med. 2016 Aug 2;13(8):e1002058. doi: 10.1371/journal.pmed.1002058 (PMC4970809; doi:10.1371/journal.pmed.1002058)
Supplement: S2 Text — (DOCX) [file pmed.1002058.s005.docx]

**S2 Text. STROBE statement**

Checklist of items that should be included in reports of observational studies

|  | Item No | Recommendation |
| --- | --- | --- |
| **Title and abstract** | 1 | (*a*) Indicate the study’s design with a commonly used term in the title or the abstract  **Within the Title on page 1:**  “Adverse renal, endocrine, hepatic and metabolic events during maintenance mood stabilizer treatment for bipolar disorder: A population based cohort study” |
|  |  | (*b*) Provide in the abstract an informative and balanced summary of what was done and what was found  **In the Methods and Findings sections of the Abstract on page 2 we wrote:**  “We conducted a propensity score adjusted cohort study using nationally representative United Kingdom electronic health records from January 1 1995 until 31 December 2013. Included patients had a diagnosis of bipolar disorder and were prescribed lithium (N=2148), valproate (N=1670) olanzapine (N=1477) or quetiapine (N=1376) as maintenance mood stabilizer treatment. Averse outcomes were chronic kidney disease, thyroid disease, hypercalcaemia, weight gain, hypertension, type 2 diabetes mellitus, cardiovascular disease and hepatotoxicity. The propensity score included important demographic, physical health and mental health predictors of drug treatment allocation.  Compared to patients prescribed lithium, those taking valproate, olanzapine and quetiapine had reduced rates of chronic kidney disease stage 3 or more severe, following adjustment for propensity score, age, calendar year and accounting for clustering by primary care practice (valproate hazard ratio [HR] 0.56; 95% confidence interval [CI] 0.45-0.69; P<0.001, olanzapine HR 0.57; 95%CI 0.45-0.71; P<0.001, quetiapine HR 0.62; 95%CI 0.47-0.80; P<0.001). Hypothyroidism was reduced in those taking valproate (HR 0.60; 95%CI 0.40-0.89; P=0.012) and olanzapine (HR 0.48; 95%CI 0.29-0.77; P=0.003), compared to those taking lithium. Rates of new onset hyperthyroidism (valproate HR 0.24; 95%CI 0.09-0.61; P=0.003, olanzapine HR 0.31; 95%CI 0.13-0.73; P=0,007) and hypercalcemia (valproate HR 0.25; 95%CI 0.10-0.60; P=0.002, olanzapine HR 0.32; 95%CI 0.14-0.76; P=0.008, quetiapine HR 0.23; 95%CI 0.07-0.73; P=0.013) were also reduced relative to lithium. However, rates of greater than 15% weight gain on valproate, olanzapine and quetiapine were higher (valproate HR 1.55; 95%CI 1.28-1.86; P<0.001, olanzapine HR 1.64; 95%CI 1.35-2.00; P<0.001, quetiapine HR 1.48; 95%CI 1.16-1.87; P<0.001) than in individuals prescribed lithium, as were rates of hypertension in the olanzapine treated group (HR 1.41, 95%CI 1.06-1.87; P=0.017). We found no significant difference in rates of chronic kidney disease stage 4 or more severe, type 2 diabetes mellitus, cardiovascular disease or hepatotoxicity. Despite estimates being robust following sensitivity analyses, limitations include the potential for residual confounding and ascertainment bias, and an inability to examine dosage effects”. |
| Introduction | | |
| Background/rationale | 2 | Explain the scientific background and rationale for the investigation being reported  **In the Introduction on page 4 we wrote:**  **“**A number of adverse effects of lithium have been identified since its use as a mood stabilizer became established in the 1970s [10], but it is only recently that they have begun to be characterised and quantified [11-15]. Lithium’s adverse effects include renal, thyroid, and parathyroid dysfunction. Lithium is also recognised to cause weight gain, but the risk of weight gain relative to other potential maintenance therapies has not been widely investigated [11]. Alternatives, such as second generation antipsychotics and valproate, have been found to be obesogenic [16], especially olanzapine, which is the most commonly prescribed antipsychotic in BPD [9]. Weight gain is associated with a number of adverse events such as hypertension, type 2 diabetes mellitus (T2DM) and cardiovascular disease (CVD) [17]. Valproate, olanzapine and quetiapine are metabolized by the liver. Valproate has been found to be associated with a high risk of asymptomatic elevated transaminases and can cause idiosyncratic hepatic failure [15, 18]. Olanzapine and quetiapine have also been associated with rare cases of hepatotoxicity [19-21]. Therefore, the balance of risks associated with maintenance mood stabilizer selection is not straightforward, and we are aware of no studies that make these comparisons across treatment options.” |
| Objectives | 3 | State specific objectives, including any prespecified hypotheses  **In the Introduction on pages 4 and 5 we wrote:**  “This study uses a large electronic patient record database to compare rates of major recognised adverse outcomes amongst individuals prescribed lithium, valproate, olanzapine or quetiapine for mood stabilization in BPD. The adverse events examined are chronic kidney disease (CKD), hypothyroidism, hyperthyroidism, hypercalcemia, weight gain, hypertension, T2DM, CVD and hepatotoxicity [15, 18]”. |
| Methods | | |
| Study design | 4 | **Present key elements of study design early in the paper**  **In the Methods on page 5 we wrote:**  **“**A population based longitudinal cohort from January 1 1995 to December 31 2013**”.** |
| Setting | 5 | Describe the setting, locations, and relevant dates, including periods of recruitment, exposure, follow-up, and data collection  **In the Methods on page 5 and 6 we wrote:**  “The Health Improvement Network (THIN) is a UK primary care database that contains anonymised patient information from routine clinical consultations [16]. The National Health Service (NHS) South-East Multicentre Research Ethics Committee approved THIN’s provision of anonymous patient data to researchers in 2003. Scientific approval for this study was obtained from the data providers Scientific Review Committee in March 2015.  THIN contained records of over 11 million people at the time of cohort extraction [22]. Included patients are broadly representative of the UK population, and physicians contributing data are representative in terms of consultation and prescribing statistics [23, 24]. Approximately 98% of the UK population is registered with a primary care physician [25]. The incidence rate of BPD in THIN has been shown to be similar to European cohorts [26] and the validity of severe mental illness diagnoses held in primary care has been established [27]. NICE guidance recommends that any patient with suspected BPD should be referred to a psychiatrist for diagnosis and treatment planning [8], as such individuals in this cohort (psychiatrist diagnosed BPD plus appropriate mood stabilizer treatment) are considered to fulfil ICD-10 criteria for BPD.  In THIN, physicians use Read codes, a hierarchical coding system, to record information [28]. These codes include diagnoses made in primary and hospital care (which map onto ICD-10 codes), symptoms, examination findings, information from specialists and test results [29]. In the UK, primary care physicians are responsible for drug prescriptions issued within the NHS, so this information is also complete and well recorded [30]. CKD, thyroid disease, T2DM, hypertension, CVD and other chronic health condition diagnoses have been validated in THIN [23].” |
| Participants | 6 | (*a*) *Cohort study*—Give the eligibility criteria, and the sources and methods of selection of participants. Describe methods of follow-up  **In the Methods on page 6 we wrote:**  “Patients with a diagnosis of BPD were included if they had at least one 28 day prescription of lithium, valproate, olanzapine or quetiapine after 1 January 1995, or after the date at which the medical records met quality assurance criteria for data entry (based on computer usage and mortality recording rates [31, 32]). Patients were excluded if they were prescribed another study drug at the start of follow-up, or in the month before this. Diagnosis of BPD could occur at any time in the patient record. For each outcome requiring haematological or biochemical confirmation for diagnosis (CKD, thyroid disease, hypercalcemia, , hepatotoxicity) patients were excluded from the primary analysis if they did not receive a specific blood test for the outcome, to reduce surveillance bias. For the weight gain outcome, patients were excluded if they did not have a baseline, or pre-treatment weight, and at least one other weight measurement. For the outcome of hyperthyroidism, patients taking thyroxine were excluded, as this can result in thyroid stimulating hormone (TSH) suppression [33]. Patients were also excluded if they had the outcome of interest at baseline (as we were interested in incident events). Therefore each outcome has a different number of patients included.” |
|  |  | (*b*) *Cohort study*—For matched studies, give matching criteria and number of exposed and unexposed  **N/A** |
| Variables | 7 | Clearly define all outcomes, exposures, predictors, potential confounders, and effect modifiers. Give diagnostic criteria, if applicable  **In the Methods on pages 6-8 we define all exposures, outcomes, and potential covariates entered into the propensity score:**  **Exposure:**  “Date of first prescription was taken as the start of exposure time. The end of the prescription was calculated from the amount prescribed and dosage instructions coded by the physician. Patients were considered to have a period of continuous prescribing if another prescription for the drug was issued within three months of the calculated end date. If this did not occur the date of stopping the study drug was the end date of the final prescription. Three months was added to this end date to account for late development of the adverse event or delayed recording. Each patient could only contribute exposure time to one of the study drugs (the first they received) and did not re-enter the cohort if they restarted the drug after more than 3 months. Patients could be prescribed other psychiatric medications but not combinations of the study drugs. If they commenced another study drug they were censored (to ensure the outcome could be assigned to a particular drug).”  **Main outcomes:**  “Outcomes of interest were: CKD stage 3 or above (or an estimated glomerular filtration rate [eGFR] of <60ml/min/1.73m^2^), CKD stage 4 or above (or an eGFR<30 ml/min/1.73m^2^) [34, 35], (if eGFR was unavailable we calculated it from available creatinine blood tests using the CKD-EPI equation [36]), hypothyroidism (or a TSH of >10mU/L) , hyperthyroidism (or a TSH<0.1mU/L) [33], hypercalcemia (adjusted calcium>2.65mmol/L) [37], >7% and >15% weight gain from baseline [38], hypertension, T2DM (or HBA1c >48mmol/mol) [39], CVD (defined as any of ischemic heart disease [IHD], myocardial infarction [MI] or cerebrovascular event [CVE]) and hepatotoxicity (or alanine transaminase [ALT] >200U/L, or aspartate aminotransferase [AST] >250U/L) [40].  Patients were followed-up until the earliest of i) the first record of the adverse event of interest, ii) the date of stopping the study drug plus three months, iii) date of switching to another study drug, iv) date of death or date of leaving the physicians practice, v) 31 December 2013.”    **And propensity score estimation:**  “A number of baseline patient characteristics were extracted from THIN. Physical and mental health conditions were considered present if referenced in patient notes and absent if they were not. If a patient had multiple entries of the same (or similar) codes, the start date of the condition was taken as the earliest date of entry.  A propensity score (PS) for each individual was estimated using variables defined *a priori,* based on existing research and clinical experience of factors influencing prescribing choice [3, 41, 42]*.* The PS is the conditional probability of receiving one study drug, rather than another, given the variables included in the model [42, 43]. Included variables were: sex, age at start of treatment with the study drug, year of entry to the cohort, ethnicity (grouped as White, Black, Asian, Mixed, other, with missing values coded as White [44]), IHD diagnosis before baseline, history of MI, history of CVE, hypertension, CKD at baseline, history of hypo or hyperthyroidism, history of liver disease or hepatotoxicity, T2DM, epilepsy, alcohol use (grouped as none/low, moderate, high/dependent), history of illicit drug use, smoking status (grouped as never-smoker, ex-smoker, current smoker), body mass index (BMI) (grouped as healthy weight, overweight (BMI 25 to 30), obese (BMI over 30)), anxiety symptoms or diagnosis before baseline, depressive symptoms or diagnosis, sleep disturbance before baseline, treatment with one of the study drugs at or before baseline and clustering by practice in which the treating physician was working. The PS was checked by comparison of covariate balance across treatments, within strata. The variables in the PS excluded the outcome variable for that particular analysis. Although PS estimation cannot remove all bias, it has been postulated to also reduce confounding from unmeasured covariates, because of their association with measured variables [45-47]. In this way, use of a PS aims to replicate a randomized experiment as closely as possible, by obtaining treatment groups with similar covariate distributions [48].” |
| Data sources/ measurement | 8* | For each variable of interest, give sources of data and details of methods of assessment (measurement). Describe comparability of assessment methods if there is more than one group  **In the Methods on page 5 we wrote:**  “THIN contained records of over 11 million people at the time of cohort extraction [22]. Included patients are broadly representative of the UK population, and physicians contributing data are representative in terms of consultation and prescribing statistics [23, 24]. Approximately 98% of the UK population is registered with a primary care physician [25]. The incidence rate of BPD in THIN has been shown to be similar to European cohorts [26] and the validity of severe mental illness diagnoses held in primary care has been established [27]. NICE guidance recommends that any patient with suspected BPD should be referred to a psychiatrist for diagnosis and treatment planning [8], as such individuals in this cohort (psychiatrist diagnosed BPD plus appropriate mood stabilizer treatment) are considered to fulfil ICD-10 criteria for BPD.  In THIN, physicians use Read codes, a hierarchical coding system, to record information [28]. These codes include diagnoses made in primary and hospital care (which map onto ICD-10 codes), symptoms, examination findings, information from specialists and test results [29]. In the UK, primary care physicians are responsible for drug prescriptions issued within the NHS, so this information is also complete and well recorded [30]. CKD, thyroid disease, T2DM, hypertension, CVD and other chronic health condition diagnoses have been validated in THIN [23].”  **If a patient had “**a diagnosis of BPD were included if they had at least one 28 day prescription of lithium, valproate, olanzapine or quetiapine after 1 January 1995, or after the date at which the medical records met quality assurance criteria for data entry (based on computer usage and mortality recording rates [31, 32]).” **Their full record was reviewed.** |
| Bias | 9 | Describe any efforts to address potential sources of bias  **Our choice of principal analytic strategy was based on reducing bias. In the** **Methods on page 6-8 we wrote:**  “For each outcome requiring haematological or biochemical confirmation for diagnosis (CKD, thyroid disease, hypercalcemia, hepatotoxicity) patients were excluded from the primary analysis if they did not receive a specific blood test for the outcome, to reduce surveillance bias.”  **And we used a propensity score approach to address confounding by indication and differences in baseline characteristics:**  “A propensity score (PS) for each individual was estimated using variables defined *a priori,* based on existing research and clinical experience of factors influencing prescribing choice [3, 41, 42]*.* The PS is the conditional probability of receiving one study drug, rather than another, given the variables included in the model [42, 43].”  “Although PS estimation cannot remove all bias, it has been postulated to also reduce confounding from unmeasured covariates, because of their association with measured variables [45-47]. In this way, use of a PS aims to replicate a randomized experiment as closely as possible, by obtaining treatment groups with similar covariate distributions [48].”  **The robustness of our results was tested using two sensitivity analyses:**  “We conducted sensitivity analyses where individuals who did not receive blood tests or weight measurements were not dropped from the cohort, and where individuals were assigned inverse probability weights (IPW) based on how likely they were to have blood test or weight records [54]. We used multiple demographic and clinical variables to predict missingness for the IPW model.” |
| Study size | 10 | Explain how the study size was arrived at  **We identified all individuals aged 16 and older in THIN with a diagnosis of bipolar disorder (at any time) treated with lithium, valproate, olanzapine or quetiapine between January 1 1995 and December 31 2013.** |
| Quantitative variables | 11 | Explain how quantitative variables were handled in the analyses. If applicable, describe which groupings were chosen and why  **In the Methods on pages 7-8 we wrote:**  **“**Outcomes of interest were: CKD stage 3 or above (or an estimated glomerular filtration rate [eGFR] of <60ml/min/1.73m^2^), CKD stage 4 or above (or an eGFR<30 ml/min/1.73m^2^) [34, 35), (if eGFR was unavailable we calculated it from available creatinine blood tests using the CKD-EPI equation [36]), hypothyroidism (or a TSH of >10mU/L) , hyperthyroidism (or a TSH<0.1mU/L) [33], hypercalcemia (adjusted calcium>2.65mmol/L) [37], >7% and >15% weight gain from baseline [38], hypertension, T2DM (or HBA1c >48mmol/mol) [39], CVD (defined as any of ischemic heart disease [IHD], myocardial infarction [MI] or cerebrovascular event [CVE]) and hepatotoxicity (or alanine transaminase [ALT] >200U/L, or aspartate aminotransferase [AST] >250U/L) [40].”  **And:**  “Included variables were: sex, age at start of treatment with the study drug, year of entry to the cohort, ethnicity (grouped as White, Black, Asian, Mixed, other, with missing values coded as White [44]), IHD diagnosis before baseline, history of MI, history of CVE, hypertension, CKD at baseline, history of hypo or hyperthyroidism, history of liver disease or hepatotoxicity, T2DM, epilepsy, alcohol use (grouped as none/low, moderate, high/dependent), history of illicit drug use, smoking status (grouped as never-smoker, ex-smoker, current smoker), body mass index (BMI) (grouped as healthy weight, overweight (BMI 25 to 30), obese (BMI over 30)), anxiety symptoms or diagnosis before baseline, depressive symptoms or diagnosis, sleep disturbance before baseline, treatment with one of the study drugs at or before baseline and clustering by practice in which the treating physician was working**”.** |
| Statistical methods | 12 | (*a*) Describe all statistical methods, including those used to control for confounding  **In the Methods on pages 8 we wrote:**  “Cox regression analyses were conducted comparing the rates of adverse events in the four treatment groups. The proportional hazards model was tested formally with analysis of Schoenfeld residuals [49]. The PS was calculated using multinomial logistic regression using drug treatment as the dependent variable and the covariates described as independent variables. The PS was then used as a linear term in a cox regression analysis that also included age, calendar year and clustering by practice [50]. In all cases this model was shown to be superior to stratifying on PS using Akaike information criterion and Bayesian information criterion [51], and was a more efficient use of data than PS matching (because no patients were excluded). To account for the competing risk of each outcome with death we plotted graphs of cumulative incidence function, adjusted for PS and age, following competing-risks regression [52, 53].We conducted sensitivity analyses where individuals who did not receive blood tests or weight measurements were not dropped from the cohort, and where individuals were assigned inverse probability weights (IPW) based on how likely they were to have blood test or weight records [54]. We used multiple demographic and clinical variables to predict missingness for the IPW model. All analyses were completed using Stata 14 [55].” |
|  |  | (*b*) Describe any methods used to examine subgroups and interactions  **N/A** |
|  |  | (*c*) Explain how missing data were addressed  **We conducted two sensitivity analyses to account for missing blood tests or weight measurements: one in which all individuals were included in the cohort, irrespective of testing, and one in which we included inverse probability weighting in the model to account for missingness.** |
|  |  | (*d*) *Cohort study*—If applicable, explain how loss to follow-up was addressed  **In the Methods on pages 7 we wrote:**  **“**Patients were followed-up until the earliest of i) the first record of the adverse event of interest, ii) the date of stopping the study drug plus three months, iii) date of switching to another study drug, iv) date of death or date of leaving the physicians practice, v) 31 December 2013.”  **In the figures, we used a competing-risks model to account for the completing risks of the outcome of interest or death:**  “To account for the competing risk of each outcome with death we plotted graphs of cumulative incidence function, adjusted for PS and age, following competing-risks regression [52, 53].” |
|  |  | (*e*) Describe any sensitivity analyses  **In the Methods on pages 8-10 we wrote:**  **“**We conducted sensitivity analyses where individuals who did not receive blood tests or weight measurements were not dropped from the cohort, and where individuals were assigned inverse probability weights (IPW) based on how likely they were to have blood test or weight records [54]. We used multiple demographic and clinical variables to predict missingness for the IPW model.**”** |

Continued on next page

| Results | | |
| --- | --- | --- |
| Participants | 13* | (a) Report numbers of individuals at each stage of study—eg numbers potentially eligible, examined for eligibility, confirmed eligible, included in the study, completing follow-up, and analysed  **In the Results on page 8 we wrote:**  **“**For each outcome 6671 individuals with BPD diagnosis were potentially included in the analysis, 2148 prescribed lithium, 1670 prescribed valproate, 1477 prescribed olanzapine and 1376 prescribed quetiapine (see S1 Figure). The characteristics of the potentially included cohort are shown in Table 1. The number of individuals included for each outcome by treatment group is shown in S1 Table**.”** |
|  |  | (b) Give reasons for non-participation at each stage  **N/A** |
|  |  | (c) Consider use of a flow diagram  **This is S1 Figure** |
| Descriptive data | 14* | (a) Give characteristics of study participants (eg demographic, clinical, social) and information on exposures and potential confounders  **In the Results on page 8 we wrote:**  **“**The characteristics of the potentially included cohort are shown in Table 1”.  **And table 1 contains this information** |
|  |  | (b) Indicate number of participants with missing data for each variable of interest  **This is shown in S1 Table and S2 Table.** |
|  |  | (c) *Cohort study*—Summarise follow-up time (eg, average and total amount)  **This is shown in Table 1 by treatment group.** |
| Outcome data | 15* | *Cohort study*—Report numbers of outcome events or summary measures over time  **This is shown in Table 2 for each oucome (row labelled events, N)** |
|  |  |  |
|  |  |  |
| Main results | 16 | (*a*) Give unadjusted estimates and, if applicable, confounder-adjusted estimates and their precision (eg, 95% confidence interval). Make clear which confounders were adjusted for and why they were included  **These are shown in Table 2 and described in the Results on page 9-10:**  **“**In unadjusted analysis and after adjustment for PS, age, calendar year and clustering by practice in which the primary care physician worked, rates of CKD stage 3 or above in individuals prescribed valproate (HR 0.56; 95%CI 0.45-0.69) olanzapine (HR 0.57; 95%CI 0.45-0.71) or quetiapine (HR 0.62; 95%CI 0.47-0.80) were reduced compared to lithium (Table 2, Figure 1). Compared to lithium, rates of hypothyroidism were reduced in those prescribed valproate (HR 0.60; 95%CI 0.40-0.89), olanzapine (HR 0.48; 95%CI 0.29-0.77), but not quetiapine (HR 0.63; 95%CI 0.38-1.05) after adjustment. Rates of hyperthyroidism were lower in those prescribed valproate (HR 0.24; 95%CI 0.09-0.61) and olanzapine (HR 0.31; 95%CI 0.13-0.73), but not quetiapine (HR 0.45; 95%CI 0.18-1.18) compared to lithium. Hypercalcemia was less common in those prescribed valproate (HR 0.25; 95%CI 0.10-0.60), olanzapine (HR 0.32; 95%CI 0.14-0.76), or quetiapine (HR 0.23; 95%CI 0.07-0.73) compared to lithium (Table 2, Figure 2). After adjustment, rates of weight gain were higher in valproate, olanzapine and quetiapine than lithium (>15% weight gain: valproate HR 1.62; 95%CI 1.31-2.01, olanzapine HR 1.84; 95%CI 1.47-2.30, quetiapine HR 1.67; 95%CI 1.24-2.20). Rates of hypertension were higher in olanzapine (HR 1.41; 95%CI 1.06-1.87) than lithium (Table 2, Figure 3). We found no significant difference in rates of CKD stage 4 or above, T2DM, cardiovascular disease, or hepatotoxicity between groups (Table 2). The median number of eGFR/creatinine and TSH blood tests per year in treatment was higher in those taking lithium than the other drugs (see S2 Table). Weight measurement and blood tests for adjusted calcium and ALT/AST were less frequent in patients prescribed lithium (see S2 Table). For outcomes in which patients had been excluded because of missing tests (CKD, hypo- and hyperthyroidism, hypercalcemia, weight gain and hepatotoxicity) sensitivity analyses including all patients resulted in reduced rate estimates compared to the primary analysis, but had little effect on HRs (see S3 Table). Sensitivity analyses using IPW suggest results from the primary analysis are robust (see S3 Table). From Schoenfeld residuals, there was no evidence against the assumption of proportional hazards for any outcome.” |
|  |  | (*b*) Report category boundaries when continuous variables were categorized  **N/A** |
|  |  | (*c*) If relevant, consider translating estimates of relative risk into absolute risk for a meaningful time period  **N/A** |
| Other analyses | 17 | Report other analyses done—eg analyses of subgroups and interactions, and sensitivity analyses  **In the Results on pages 10 we wrote:**  “For outcomes in which patients had been excluded because of missing tests (CKD, hypo- and hyperthyroidism, hypercalcemia, weight gain and hepatotoxicity) sensitivity analyses including all patients resulted in reduced rate estimates compared to the primary analysis, but had little effect on HRs (see S3 Table). Sensitivity analyses using IPW suggest results from the primary analysis are robust (see S3 Table).” |
| Discussion | | |
| Key results | 18 | Summarise key results with reference to study objectives  **Objectives:**  **to compare rates of major recognised adverse outcomes amongst individuals prescribed lithium, valproate, olanzapine or quetiapine for mood stabilization in BPD. The adverse events examined are chronic kidney disease (CKD), hypothyroidism, hyperthyroidism, hypercalcemia, weight gain, hypertension, T2DM, CVD and hepatotoxicity [15, 18].**  **Key results:**  **Compared to patients prescribed lithium, those taking valproate, olanzapine and quetiapine had reduced rates of chronic kidney disease stage 3 or more severe, following adjustment for propensity score, age, calendar year and accounting for clustering by primary care practice (valproate hazard ratio [HR] 0.56; 95% confidence interval [CI] 0.45-0.69, olanzapine HR 0.57; 95%CI 0.45-0.71, quetiapine HR 0.62; 95%CI 0.47-0.80). Hypothyroidism was reduced in those taking valproate (HR 0.60; 95%CI 0.40-0.89) and olanzapine (HR 0.48; 95%CI 0.29-0.77), compared to those taking lithium. Rates of new onset hyperthyroidism (valproate HR 0.24; 95%CI 0.09-0.61, olanzapine HR 0.31; 95%CI 0.13-0.73) and hypercalcemia (valproate HR 0.25; 95%CI 0.10-0.60, olanzapine HR 0.32; 95%CI 0.14-0.76, quetiapine HR 0.23; 95%CI 0.07-0.73) were also reduced relative to lithium. However, rates of greater than 15% weight gain on valproate, olanzapine and quetiapine were higher (valproate HR 1.55; 95%CI 1.28-1.86, olanzapine HR 1.64; 95%CI 1.35-2.00, quetiapine HR 1.48; 95%CI 1.16-1.87) than in individuals prescribed lithium, as were rates of hypertension in the olanzapine treated group (HR 1.41, 95%CI 1.06-1.87). We found no significant difference in rates of chronic kidney disease stage 4 or more severe, type 2 diabetes mellitus, cardiovascular disease or hepatotoxicity.** |
| Limitations | 19 | Discuss limitations of the study, taking into account sources of potential bias or imprecision. Discuss both direction and magnitude of any potential bias  **In the Discussion on pages 14-15 we wrote:**  **“**The major strength of this study, beyond size and length of follow-up, is the direct comparison between BPD maintenance mood stabilizer treatment options for a number of adverse effects. The use of electronic health records also means it is possible to adjust for a number of demographic and physical health characteristics that may have influenced the clinician’s decision to treat with a particular medication or potentially confound the relationship between treatment and adverse outcome. Despite including numerous variables in the PS, it is possible that residual confounding remained, especially as those prescribed lithium were older and were more likely to have taken the drug previously, perhaps reflecting a more chronic illness course. It may be that important patient or clinician features were not captured by the score, and despite the balance of observed covariates we cannot confirm balance of unobserved covariates [66, 67]. We were also unable to consider dosage differences across the different treatment groups in this analysis. Periods of lithium toxicity may be particularly important with regards to developing renal failure and we were unable to capture this information from the available data. Missing data can be a problem in studies utilising electronic patient records, especially as there may be a clinical reason why information is missing. Because of the way outcomes were defined T2DM, cardiovascular disease and diagnoses of hypertension had no missing data, and no covariates in the PS had missing values.  Patients prescribed lithium had no more physician contacts than those taking other mood stabilizer medication. In individuals that *ever* received tests during treatment exposure, testing frequency was similar in all study drugs for adjusted calcium, liver function and weight (S2 Table). Frequency of testing renal and thyroid function was higher in those taking lithium, which reflects the guidance for monitoring [8]. Patients prescribed lithium were also more likely to have at least one renal function, thyroid function, calcium or liver function test compared to patients taking other drugs. This is likely to be due to both drug related indications for monitoring, and the longer drug exposure seen in those taking lithium. IPW sensitivity analysis to account for this difference did not alter our conclusions (S3 Table). In the primary analysis the likely effect of this differential missingness would be to reduce the hazard ratios for lithium compared to the other drugs, relative to their true values, as blood tests in the non-lithium group are more likely to be related to clinical symptoms than monitoring guidance (for instance, this is likely to represent an underestimation of the true hypercalcemia hazard ratio for lithium versus other drugs). The median number of weight measurements was similar in each group suggesting detection of weight gain was not related to differential monitoring. The sensitivity analysis including individual’s irrespective of blood tests produced similar adjusted hazard ratios the primary analysis for each outcome, but often with reduced incidence of the outcome in each treatment group (S3 Table). This analysis may more accurately reflect testing occurring because of clinical indication.” |
| Interpretation | 20 | Give a cautious overall interpretation of results considering objectives, limitations, multiplicity of analyses, results from similar studies, and other relevant evidence  **In the Discussion on page 15-16 we wrote:**  “Lithium remains an important treatment option for individuals with BPD. However there is clear evidence that that its use is associated with a number of adverse events. These risks need to be offset with the potentially superior effectiveness and anti-suicidal benefits of the drug compared to other treatment options [5, 68]. It is also true that other recommended maintenance treatments can have serious side effects, often related to weight gain, and are not suitable for use in certain patient groups (such as the contraindication of valproate in women of childbearing potential [3]).  Assiduous monitoring of patients prescribed lithium should ameliorate some risk associated with effects on renal physiology and endocrine systems. Given the need to balance an array of risks and benefits, an individualised and collaborative approach to treatment choice is likely to be most appropriate. To achieve this, further research identifying patient characteristics that are risk factors for specific side effects, and an understanding of the risks and benefits of stopping treatment in those who experience adverse effects is necessary.” |
| Generalisability | 21 | Discuss the generalisability (external validity) of the study results  **In the Methods on pages 5 we wrote:**  “Included patients are broadly representative of the UK population, and physicians contributing data are representative in terms of consultation and prescribing statistics” |
| Other information | | |
| Funding | 22 | Give the source of funding and the role of the funders for the present study and, if applicable, for the original study on which the present article is based  **Joseph F Hayes is supported by a Medical Research Council Population Health Scientist Fellowship (grant code: MR/K021362/1). Funders had no role in the study design, in the collection of data or in the analysis and interpretation of data.** |
