# Supplementary material for: Adverse Renal, Endocrine, Hepatic, and Metabolic Events during Maintenance Mood Stabilizer Treatment for Bipolar Disorder: A Population-Based Cohort Study
Source: PLoS Med. 2016 Aug 2;13(8):e1002058. doi: 10.1371/journal.pmed.1002058 (PMC4970809; doi:10.1371/journal.pmed.1002058)
Supplement: S3 Text — (DOCX) [file pmed.1002058.s006.docx]

**S3 Text. Prospective analysis plan**

# Rates of side effects with recommended maintenance treatment for bipolar disorder: A UK primary care cohort study

**The following was agreed on 21.07.15 by JFH, LM, KW, JRG, MK, DPJO**

# Scientific rationale

From September 2014 the updated NICE guidance was to use lithium as first-line maintenance treatment in bipolar disorder (BPAD). However in clinical practice this is often avoided due to lithium’s side effect profile compared to other recommended drug treatments.

The incidence rates of side effects of lithium are poorly defined, as are the rates of side effects of other commonly used maintenance medications. It is recognised that lithium may increase risk of renal, thyroid and calcium abnormalities, valproate may increase the risk of liver failure and weight gain and olanzapine and quetiapine are associated with weight gain, diabetes and cardiovascular disease.

# Objectives

To determine incidence rates of side effects associated with drugs used for maintenance treatment for BPAD:

1. Lithium – chronic kidney disease, thyroid disease, calcium abnormalities
2. Valproate – liver disease, weight gain
3. Olanzapine and quetiapine – cardiovascular disease, diabetes mellitus, weight gain, hypertension

To potentially identify risk factors for developing specific side effects

# Methods

## Study design and setting

Cohort study using data from THIN, a UK primary care database representative of the UK population.

## Participants

Individuals over the age of 16 who have a diagnosis of BPAD at any time and are prescribed one of:

1. Lithium
2. Valproate
3. Olanzapine
4. Quetiapine

Individuals are considered exposed from the latest of i) date that they receive first prescription for the drug, ii) 1 January 1995, iii) ACU and AMR date.

Individuals leave the cohort at the earliest of i) date of first record of the outcome of interest, ii) date of stopping the study drug or receiving another of the study drugs + 3 months (to account for delay in development or recording of side effects), iii) date of death, iv) 31 December 2013.

Individuals will be considered continuously exposed if there is a gap of <3 months between prescriptions. Individuals will not re-enter once they have ceased their first period of treatment. If individuals receive >1 of the study drugs concurrently from the start of follow-up they will be excluded. Individuals can receive any other psychotropic treatment during the exposure period.

## Exclusions

For each analysis individuals who have the outcome of interest at baseline will be excluded.

## Main outcomes

1. >Stage 3 CKD (or eGFR<60)
2. >Stage 4 or stage 5 CKD (or eGFR<30)
3. Hypothyroidism (thyrotropin>6.0mU/L)
4. Hyperthyroidism (thyrotropin <0.2mU/L)
5. Adjusted calcium concentration >2.65 mmol/L
6. DM (or HBA1C>48mmol/L)
7. Cardiovascular disease (MI, CVE, IHD)
8. Weight gain (7% and 15% increase of body weight)
9. Hypertension
10. Liver failure (consider how to define ie ALT, ASTm PT, INR)

## Main covariates of interest

A propensity score (PS) will be built using relevant covariates related to treatment allocation, including (but not limited to):

1. Age at treatment allocation
2. Sex
3. Year of treatment allocation
4. Presence of any other study outcomes at baseline (ie physical health)
5. Ethnicity
6. Smoking status
7. Alcohol use
8. BMI
9. Hypertension
10. Anxiety symptoms
11. Depressive symptoms
12. Sleep disturbance
13. Diagnosis of BPAD before baseline
14. Treatment with study drug before baseline

PS will be refined and checked for balance across treatment groups.

## Solution to missing blood tests required for outcome

**Issue:** Individuals are more likely to have a blood test (and therefore an abnormal result) if they are prescribed a drug which is an indication for GP performing that blood test (i.e lithium adverse event is hypothyroidism, therefore more TSH blood tests).

**Approach:** Only include individuals with at least 1 of the blood tests after entry to the study.

## Analysis plan

Incidence rates of all outcomes will be determined by treatment group. Cox regression analyses will be completed using the PS and time changing variables (age and calendar year) to calculate adjusted hazard ratios for each outcome. We will explore the suitability and benefits of using the PS in different ways (i.e in regression, stratification, matching or weighting). The most suitable model will be presented. KM plots for each outcome will be presented with a suitable exposure period as the x-axis.

# Deviations from this plan

**The following was agreed on 13.10.15 by JFH, LM, KW, MK, DPJO**

1. Cut-offs for cases redefined based on most appropriate international guidelines:
   1. Hypothyroidism TSH>10mU/L
   2. Hyperthyroidism TSH<0.1mU/L (and no treatment for hypothyroidism)
   3. Liver failure AST>250U/L or ALT>200U/L
2. Additional eGFRs calculated using CKD-EPI equation and creatinine blood test results following discussion with renal physician.

**The following was agreed on 03.03.16 by JFH, LM, KW, JRG, MK, DPJO**

1. KM plots dropped at suggestion of reviewer as these fail to account for the competing risk of death. Plots from competing-risks regression presented as an alternative. However, Cox regression results still presented in tables as subdistribution HRs cannot be interpreted as HRs.
2. Sensitivity analyses added at request of reviewers:
   1. All individuals included (i.e not dropped if no blood test)
   2. Inverse probability weighting based on missing blood tests
